# Physician resilience and perceived quality of care among medical doctors with training in psychosomatic medicine during the COVID-19 pandemic: a quantitative and qualitative analysis

**DOI:** 10.1186/s12913-024-10681-1

**Published:** 2024-02-27

**Authors:** Christian Fazekas, Maximilian Zieser, Barbara Hanfstingl, Janika Saretzki, Evelyn Kunschitz, Luise Zieser-Stelzhammer, Dennis Linder, Franziska Matzer

**Affiliations:** 1 Department of Psychiatry, Psychosomatics and Psychotherapy, Division of Medical Psychology, Psychosomatics and Psychotherapy, Medical University of Graz, Auenbruggerplatz 3, 8036 Graz, Austria; 2 Independent researcher, Vienna, Austria; 3 Department of Psychology, University of Klagenfurt, Klagenfurt, Austria; 4 Department of Psychology, University of Graz, Graz, Austria; 5 II. Medical Department for Cardiology, Hanusch Hospital, Vienna, Austria; 6 Karl Landsteiner Institute for Scientific Research in Clinical Cardiology, Hanusch Hospital, Vienna, Austria; 7 Department of Psychosocial, Psychosomatic and Psychotherapeutic Medicine, Austrian Medical Association, Vienna, Austria; 8 Ben Gurion University of the Negev, Beer-Sheva, Israel

**Keywords:** Continuing medical education, COVID-19 pandemic, Resilience, Physicians, Quality of care, Psychosomatic medicine

## Abstract

**Background:**

At an individual level, physician resilience protects against burnout and against its known negative effects on individual physicians, patient safety, and quality of care. However, it remains uncertain whether physician resilience also correlates with maintaining a high level of healthcare quality during crises such as a pandemic. This study aimed to investigate whether higher resilience among physicians, who had received training in resilience-related competences in the past, would be associated with higher quality of care delivered during the COVID-19 pandemic.

**Methods:**

This study enrolled physicians working in family medicine, psychiatry, internal medicine, and other medical specialties, who had obtained at least one of three consecutive diplomas in psychosomatic medicine in the past. Participants completed a quantitative and qualitative anonymous online survey. Resilience was measured using the Connor-Davidson Resilience Scale, and healthcare quality was assessed through single-item quality indicators, including perceived quality of care, professional autonomy, adequate time for patient care, and job satisfaction.

**Results:**

The study included 229 physicians (70 males/159 females) with additional training in psychosomatic medicine, working in family medicine (42.5%), psychiatry (28.1%), internal medicine (7.0%), or other medical specialties (22.4%). Participants represented four intensity levels of training background (level 1 to level 4: 9.2%, 32.3%, 46.3%, and 12.2% of participants). Training background in psychosomatic medicine was positively associated with resilience (B = 0.08, SE = 0.04, *p* <.05). Resilience and training background independently predicted perceived quality of care, even after controlling for variables such as own health concerns, involvement in the treatment of COVID-19 patients, financial strain, percentage of working hours spent on patient care, age, and gender (resilience: B = 0.33, SE = 0.12, *p* <.01; training background: B = 0.17, SE = 0.07, *p* <.05). Both resilience and training background predicted job satisfaction (resilience: B = 0.42, SE = 0.12, *p* <.001; training background: B = 0.18, SE = 0.07, *p* <.05), while resilience alone predicted professional autonomy (B = 0.27, SE = 0.12, *p* <.05). In response to an open question about their resources, resilient physicians more frequently reported applying conscious resilient skills/emotion regulation (*p* <.05) and personal coping strategies (*p* <.01) compared to less resilient medical doctors.

**Conclusion:**

Physician resilience appears to play a significant role in the perceived quality of patient care, professional autonomy, and job satisfaction during healthcare crises.

**Supplementary Information:**

The online version contains supplementary material available at 10.1186/s12913-024-10681-1.

## Introduction

Resilience is the ability of an individual to respond to stress and adversity in an adaptive way such that goals are achieved at minimal psychological and physical costs and mental well-being rapidly ”bounces back” [1]. As resilience can counteract negative effects of workplace stress, the concept of physician resilience has emerged in response to high prevalence rates of physician burnout and physician stress [2–4], which seem to have substantially increased during the COVID-19 pandemic [5–7].

Negative effects of burnout include personal consequences, such as depression, substance use, disrupted relationships and suicide; burnout impacts on professional attitude and performance, with subsequent lower quality of care, higher rates of medical errors, decreased patient satisfaction, decreased productivity, and increased clinician turnover [8–12]. A large systematic review and meta-analysis including 170 observational studies reported doubled odds of patient safety incidents and almost fourfold odds of decreased job satisfaction associated with physician burnout, in addition to other aspects of reduced quality of care and increased career disengagement [13].

The COVID-19 pandemic imposed high stress levels on frontline healthcare workers accompanied by reduced professional quality of life and increased risk for the onset of depression and anxiety disorders [14, 15]. Consequently, building psychological resilience emerged as an essential focus in response to these challenges, as it may serve as a protective factor against the risk of developing burnout and its negative consequences on individuals and the health care system [14, 16]. Higher personal resilience was found to be associated with lower stress levels and lower levels of anxiety, COVID-19 related fear, depression, fatigue, and sleep disturbances during the COVID-19 pandemic [17–19]. Besides the well-known positive effects of resilience on physicians’ well-being, there is some evidence suggesting that it could also protect against detrimental effects of a health care crisis by maintaining a high level of health care quality.

Health care quality is the degree to which health services for individuals and populations increase the likelihood of desired health outcomes [20]. One study reported that physician resilience contributed to maintaining health care quality during a financial crisis in the health care system in Portugal [21]. Another study reported an association between resilience among nurses and perceived quality of care during the COVID-19 pandemic [22]. A further study *on health care workers* demonstrates the influence of resilience in reducing burnout during the pandemic [23]. However, studies investigating specifically an influence of physician resilience on perceived quality of delivered care during this pandemic seem to be widely lacking.

The main aim of this study was (1) to evaluate an assumed association of physician resilience and training background in psychosomatic medicine with perceived quality of care during the COVID-19 pandemic while controlling for potential confounding variables (age, gender, number of patients, treatment of COVID-19 patients, own health concerns, financial strains). We also aimed at (2) investigating the individual and combined contributions of resilience and training background to professional autonomy, sufficient time for patient care and job satisfaction as secondary dependent variables. Participants in the study were enrolled among medical doctors who had participated in a graded resilience-related training, which is an integral part of a consecutive three tier continuing medical education (CME) program in psychosomatic medicine in Austria [24]. The participants’ selection allowed us to look for (3) a possible association of the consecutive training levels with reported physician resilience. Finally, the qualitative part of the study aimed at (4) exploring self-reported challenges and resources during the COVID-19 pandemic and (5) analyzing differences according to high and low resilience subgroups and gender.

## Methods

### Sample and procedure

In this study, we aimed at evaluating physician resilience, training background, and work-related experiences during the COVID-19 pandemic. Medical specialists with an additional certified training background in psychosomatic medicine were chosen as study cohort as these long-term CME programs also include resilience building and preventative measures and techniques. In Austria, there are three consecutive levels of long-term training in psychosomatic medicine with a duration between 1 year (PSY-1, Diploma for Psychosocial Medicine), additional 2 years (PSY-2, Diploma for Psychosomatic Medicine) and additional 3 years (PSY-3, Diploma for Psychotherapeutic Medicine). Regarding personal development, the training involves supervision, Balint group work and participation in self-awareness groups. It also promotes self-management, communication skills and psychotherapeutic techniques; the third training level is leading to full psychotherapeutic competence [24]. For the purposes of the present study, a fourth level termed PSY-4 was defined for participants who reported an additional certification in the psychosomatic and psychotherapeutic field beyond PSY-3.

The study was approved by the ethics committee of the Medical University of Graz (32-534ex 19/20). Informed consent was obtained from all participants before data collection. The study was performed via an online-survey with the intention to reach out to all 2807 Austrian medical doctors with additional training background in psychosomatic medicine. A response rate of about 10% was assumed. As inclusion criteria, participants had to be approved in their specific medical field and they had to be currently working in their profession. In order to reach the target group, physicians received an invitation to participate in the study via personal email or within a newsletter by their corresponding Federal Association of Medical Doctors and the Austrian Society for Psychosomatics and Psychotherapeutic Medicine (ÖGPPM). By this way, they also received a token created via the online survey tool LimeSurvey in order to guarantee that only members of the target group could participate. Anonymized quantitative and qualitative data were collected during July and September 2020, the data collection took therefore place shortly after the first COVID-19 lockdown period in Austria (March/ April 2020).

### Measures

#### Resilience

The trait resilience was assessed using the German 10-item-version of the Connor-Davidson Resilience Scale (CD-RISC) [25, 26]. The German translation of the unidimensional instrument, as applied in this study, has shown very good psychometric properties with high internal consistency (Cronbach’s α = 0.84). The statements of the self-report assessment are rated on a 5-point Likert scale (example item: “Even if there are obstacles, I believe I can achieve my goals”), adding up to a total sum value ranging from 10 to 50. Item values were averaged, resulting in a scale ranging from 1 to 5, with higher values indicating higher resilience.

#### Perceived quality of care, professional autonomy, time for patient care, and job satisfaction

We applied four one-item outcome measures that represent quality indicators for health care systems and health care quality, and have been used and validated previously [27–30]. These outcome variables encompassed statements about perceived quality of care (“It is possible to provide high quality care to all of my patients”), adequate time for patient care (“I have adequate time to spend with my patients during a typical patient visit”) and professional autonomy (“I have the freedom to make clinical decisions that meet my patients’ needs”). Items were scored on a five-point scale ranging from 1 = ‘strongly disagree’ to 5 = ‘strongly agree’.

Job satisfaction was measured by the question “On the whole, how satisfied were you/are you with your job?” This item was scored on a five-point scale ranging from 1 = ‘very dissatisfied’ to 5 = ‘very satisfied’ [27, 31].

Answers to each of these four outcome measures were given in a tabular format. The assessment of the questions was only performed once during the time after the first lockdown, however, these four questions were answered not only for today but also retrospectively for two periods of time: the time before the COVID-19 epidemic, and the time during the initial lockdown in Austria.

#### Sociodemographic and professional characteristics

In addition to the items of the CD-RISC and the four one-item outcome measures mentioned above, further items on sociodemographic data, the medical specialty, the training background in psychosomatic medicine and work-related data were developed for this study. An English language version of the newly developed parts of the survey is provided as supplemental material in the Additional File 1. We asked participants to indicate the percentage of total work time typically spent on direct patient care. In addition, we inquired about the average number of hours worked during three specific time periods (before the COVID-19 pandemic, during the initial lockdown from mid-March to late April 2020, and at the time of questionnaire completion). We additionally asked if COVID-19 patients were treated during the lockdown (yes/no), if there were health concerns for one´s own health during the lockdown (4-point rating scale ranging from “yes” to “no”) and if there was a financial strain in the current situation (7-point rating scale ranging from “0” = no agreement, to “6” = full agreement).

#### Qualitative questions

Challenges during the crisis were assessed by the open question “Please indicate the three most difficult job-related challenges during the COVID-19 crisis”. Resources were measured by the open question “Please indicate three things that helped you most in your professional practice during the COVID-19 crisis”.

See Additional File 1: Survey.

### Statistical analysis

Before statistical analysis, all collected data were controlled before processing. Data collection was carried out with the online survey tool LimeSurvey hosted by the Medical University of Graz. Subsequently, we stored, processed, and analyzed data at the server park of the Medical University of Graz which is reliably protected from external access.

As descriptive statistics, we first calculated mean values of all our variables of interest across participants’ level of training in psychosomatic medicine. As there appeared to be a distinct positive linear trend in dependent variables across the four training groups, we subsequently used training level as a continuous variable in the analysis. These descriptive data are presented as supplemental material in the Additional File. Moreover, we present simple correlations between our variables of interest.

For our main results, we used linear multiple regressions on the four dependent variables quality of care, professional autonomy, adequate time for patient care (hereinafter time for patients), and job satisfaction. The dependent variables entered the model as participant means of the three measurements, e.g., quality of care in these regressions enters as each participant’s mean of responses concerning before, during, and after the COVID-19 lockdown. In addition, we also conducted linear regressions with resilience as the dependent variable. As one of the covariates, we used concerns about one’s health due to COVID-19 during the lockdown (health concerns). Previous studies showed that fear of infection can influence willingness to provide patient care [32]. Health concerns may thus also play an important role for the relationships assessed here. Further, we controlled for five additional variables that may act as confounders: involvement in COVID-19 patients treatment, financial strain as perceived at the time of completing the survey, the percentage of working time spent on patient care, age (as a continuous variable, measured in 6 categories from under 30 to over 69, see Table 1) and gender.

**Table 1:** Sociodemographic and work-related characteristics of the sample (*N* = 229)

		n (%)
**Gender**	Male	70 (30.6)
	Female	159 (69.4)
**Age group**	< 30 years	1 (0.4)
	30–39 years	10 (4.4)
	40–49 years	68 (29.7)
	50–59 years	88 (38.4)
	60–69 years	52 (22.7)
	> 69 years	10 (4.4)
**Medical specialty**	Family medicine/general practice	97 (42.5)
	Gynecology/Obstetrics	11 (4.8)
	Internal medicine	16 (7.0)
	Pediatrics	3 (1.3)
	Child- and Youth Psychiatry	8 (3.5)
	Neurology; Neurology and Psychiatry	5 (2.2)
	Psychiatry and psychotherapeutic Medicine	64 (28.1)
	Other specialties	25 (10.5)
**Place of work**	Hospital / outpatient clinic	47 (20.5)
	Other (outside hospital, e.g. physician office)	182 (79.5)
**Levels of training in psychosomatic medicine**	Level 1	21 (9.2)
	Level 2	74 (32.3)
	Level 3	106 (46.3)
	Level 4	28 (12.2)
**COVID-19 patients**	Yes	62 (27.1)
	No	127 (55.5)
	Unsecure	39 (17.0)
	Missing	1 (0.4)
**Own health concerns**	Mean (SD); Median (Range)	2.69 (1.01); 3.0 (1–4) 58 (
	Yes (= 1)	35 (15.3) 58 (
	Rather yes (= 2)	58 (25.3)
	Rather no (= 3)	78 (34.1)
	No (= 4)	57 (24.9)
	Missing	1 (0.4)
**Financial strain** ^**1**^	Mean (SD); Median (Range)	2.07 (1.21); 2.0 (1–5)
**Patient care (%)** ^**2**^	Mean (SD); Median (Range)	72.1 (22.11); 80.0 (0-100)
**Working hours before** ^**3**^	Mean (SD); Median (Range)	36.05 (14.73); 36.0 (3–80)
**Working hours during** ^**3**^	Mean (SD); Median (Range)	27.86 (19.10); 25.0 (0–80)
**Working hours after** ^**3**^	Mean (SD); Median (Range)	35.09 (16.36); 35.0 (0–90)

^1^Financial strain was measured on a 7-point rating scale ranging from “0” = no agreement, to “6” = full agreement; *n* = 214. ^2^percentage of total work time typically spent on direct patient care; *n* = 229. ^3^average number of hours worked during three specific time periods: before the COVID-19 pandemic, during the initial lockdown, and after, at the time of questionnaire completion; *n* = 229

As to conditions for applying linear models, we tested for appropriate model specification, particularly using conventional linear models, using the Ramsey Regression Equation Specification Error Test, for normality using the Shapiro-Wilk test, and for homoscedasticity using the Breusch-Pagan test. We found no violations of assumptions concerning linearity or homoscedasticity with all *p* >.05. Concerning normality, the Shapiro-Wilk test was significant for all four models, indicating non-normal distributions of residuals. However, considering the samples size of 214 participants, we are confident that conventional linear regressions are appropriate as opposed to alternative methods [33].

By design, the previously mentioned regressions on the participant means are not able to capture differences between the three time periods. We thus supplemented the analyses using repeated measures ANOVAs to account for the multiple responses regarding the dependent variables, once concerning the time of completing the questionnaire, once concerning the time of the lockdown, and once concerning the time before the pandemic.

Importantly, results of these rANOVAs can be differentiated into “between-subject” effects, which represent the effect of our independent variables on the mean of the three measurements per dependent variable exactly as captured by the regressions described above, and “within-subject” effects. In our case, the within-subject effects represent differences between the time periods which the three measurements represent. We also tested for interaction effects between the time period and our main independent variables. Results from all repeated measures ANOVAs are presented in the Additional File.

The aforementioned statistical analyses were conducted using R version 4.2.2 and the packages lme4 version 1.1–31 [34] as well as lmerTest version 3.1-3 [35] to compute mixed models and rANOVA parameters, respectively.

Qualitative data analysis (QDA) and an additional frequency analysis were conducted using the statistical software program MAXQDA 2022. One of the authors (FM) developed the initial coding framework including code definitions and examples, employing a thematic coding approach [36]; thus, the categories were drawn inductively out of the answers. After critical discussion within the study group and adaption of the initial coding framework, a second rater (JS) independently coded the texts based on the existing coding framework.

After the second coding, interrater reliability was calculated using Cohen’s Kappa statistic and reached 0.95 (range 0.83-1.0), with a Kappa of at more than 0.8 representing almost perfect match between two raters [37]. Finally, any discrepancies in coding (texts with a match below a Kappa of 0.9 between the two raters) were resolved through critical discussion until consensus was guaranteed. For analyzing differences in self-reported challenges and resources, physician resilience (high vs. low resilience) and gender (male / female) were utilized as independent variables and the categories of QDA as dependent variables. Groups with high vs. low resilience were determined by median split of the CD-RISC. Differences between the categories were calculated by Chi-Square tests using IBM SPSS 26; a *p*-value of less than 0.05 was considered significant.

## Results

### Sample description

The final sample consisted of *N* = 229 respondents (response rate: 8.2%), with a higher proportion of female participants (69.4%). Physicians were working in the fields of family medicine (42.5%), psychiatry (28.1%), internal medicine (7.0%), and other medical specialties (22.4%), with most of them working outside hospitals (79.5%) and spending most of their working time on direct patient care (72,1%). Around 27% had treated COVID-19 patients and around 40% had health concerns for their own health. Mean working hours were lower during the initial lockdown, but had reached the initial level at the time of questionnaire completion. Perceived financial strain of the sample was rather low (mean = 2 on a scale from 0 to 6). Concerning the mean resilience of study participants, we find a mean of 4.1 which corresponds to a value of 31 on a scale of 0 to 40 and is within the typical range of an adult population [38].

Details of all sociodemographic and work-related sample characteristics are presented in Table 1.

### Quantitative results

#### Correlations of training level and resilience with outcome variables

For a first evaluation of the investigated associations, simple correlation coefficients were calculated using the participant means of the outcome measures regarding the three time periods. Correlations between training levels in psychosomatic medicine, resilience, all outcome measures (perceived quality of care, professional autonomy, time for patients, job satisfaction), and control variables (own health concerns, involvement in the treatment of COVID-19 patients, financial strain, percentage of working hours spent on patient care, age, and gender) are shown in Table 2. Correlation coefficients indicate strong, positive correlations (0.47 to 0.68) between the four outcome measures and positive correlations between the outcomes and training levels as well as resilience, with the exception of resilience and time for patients. Moreover, we found a positive association between psychosomatic training and resilience, and a negative association between resilience and health concerns. With regard to control variables, we found no association between the outcome variables with gender or the proportion of work time spent on patient care.

**Table 2:** Correlations of training level and resilience with outcome variables and control variables

Variables	1.	2.	3.	4.	5.	6.	7.	8.	9.	10.	11.
1. Training	-										
2. Resilience	0.14*	-									
3. Quality of care^a^	0.22**	0.22***	-								
4. Professional autonomy^a^	0.19*	0.19**	0.68***	-							
5. Time for patients^a^	0.16*	0.09	0.60***	0.61***	-						
6. Job satisfaction^a^	0.23***	0.29***	0.53***	0.49***	0.47***	-					
7. Health concerns^b^	-0.03	-0.20**	-0.08	-0.11	-0.12	-0.19**	-				
8. Treated Cov. patients^b^ (No = 0/Yes = 1)	-0.12	0.08	-0.14*	-0.14*	-0.23***	-0.16*	0.02	-			
9. Financial strain^b^	0.11	-0.06	-0.18**	-0.16*	-0.09	-0.20**	0.04	0.01	-		
10. Patients^c^ (% of work)	-0.06	0.06	0	-0.06	-0.03	0.01	0.02	0.03	0.07	-	
11. Age	0.32***	0.02	0.17*	0.13	0.09	0.19**	0.03	-0.12	0.02	-0.02	-
12. Gender (M = 0/F = 1)	0.00	0.00	-0.07	0.02	0.04	-0.04	0.08	-0.02	0.01	0.13*	0.00

^a^ mean value of before, during and after the lockdown; ^b^ lower sample size (*N* = 214); ^c^ percentage of total work time typically spent on direct patient care; *p <.05, **p <.01, ***p <.001

#### Resilience and training levels as predictors of perceived quality of care and further quality indicators

As our main outcomes of interest, we used the dependent variables perceived quality of care, professional autonomy, time for patients, and job satisfaction, each measured three times reflecting participants assessment before the COVID-19 pandemic, during the first lockdown, and when completing the survey. In the regressions presented here, we averaged the three time periods for each participant.

As the main predictors, we used resilience and training level in psychosomatic medicine, which both entered as continuous variables. As further covariates, we used concerns about one’s health due to COVID-19 during the lockdown (health concerns), involvement in COVID-19 patients treatment, financial strain as perceived at the time of completing the survey, the percentage of working time spent on patient care, age (as a continuous variable, measured in 6 categories from under 30 to over 69, see Table 1) and gender.

Results from the regressions are presented in Table 3. We found that resilience and psychosomatic training both significantly contribute to explaining the perceived quality of care provided to patients. Moreover, we found that resilience and psychosomatic training are positively associated with job satisfaction. Furthermore, resilience is also significantly associated with professional autonomy.

**Table 3:** Regressions on the participant means of quality of care, professional autonomy, time for patients, and job satisfaction

	Quality of care B (SE)	Professional autonomy B (SE)	Time for patients B (SE)	Job satisfaction B (SE)
Training	0.17* (0.07)	0.13 (0.07)	0.12 (0.08)	0.18* (0.07)
Resilience	0.33** (0.12)	0.27* (0.12)	0.13 (0.13)	0.42*** (0.12)
Control variables:				
Health concerns	0.01 (0.05)	-0.04 (0.05)	-0.05 (0.06)	-0.10 (0.05)
Treated Cov. Patients	-0.26* (0.12)	-0.24 (0.12)	-0.44** (0.13)	-0.33** (0.12)
Financial strain	-0.13** (0.04)	-0.11* (0.04)	-0.07 (0.05)	-0.14** (0.04)
Patients	0.00 (0.00)	0.00 (0.00)	0.00 (0.00)	0.00 (0.00)
Age	0.09 (0.06)	0.05 (0.06)	0.01 (0.07)	0.10 (0.06)
Gender	-0.16 (0.12)	0.03 (0.12)	0.06 (0.13)	-0.12 (0.12)

The sample size for this calculation is *N* = 214. This is a reduced number of participants in comparison to the overall number of participating physicians because health concerns and financial strain are included as independent variables and were completed by fewer participants. *p <.05, **p <.01, ***p <.001

In order to assess the relevance of the investigated time periods on the reported results, we conducted rANOVAs as additional analyses. While our main findings (i.e., the between-subject effects described above) remain unchanged, there are significant differences between the three periods (i.e., within-subject effects) as all four dependent variables were perceived as significantly lower during the lockdown. Moreover, we find interactions between time period and psychosomatic training for professional autonomy and for time for patients. These analyses are presented in detail as supplemental material in the Additional File 2.

See Additional File 2: Training levels in psychosomatic medicine and differences in time periods.

#### Association of training levels with reported physician resilience

As shown in the correlation table (Table 2), training levels in psychosomatic medicine and resilience have a small but significantly positive correlation. To test whether this relationship holds when control variables are included, we conducted regressions with resilience as the dependent variable, once without and once with control variables (see Table 4). We found that psychosomatic training is positively associated with resilience when the set of control variables was included.

**Table 4:** Regressions of training on resilience

DV: Resilience	B (SE)	B (SE)
Training	0.08* (0.04)	0.08* (0.04)
Control variables:		
Health concerns	-	-0.09** (0.03)
Treated Cov. Patients	-	0.07 (0.07)
Financial strain	-	-0.03 (0.03)
Patients	-	0.00 (0.00)
Age	-	-0.01 (0.03)
Gender	-	-0.01 (0.07)

The sample size for the first column is *N* = 229, for the second *N* = 214. The latter is reduced because health concerns and financial strain are included as independent variables and were completed by fewer participants. *p <.05, **p <.01

Regarding quality of care and the other outcome variables, resilience appeared to act both as predictor and as a weak mediator, capturing a small proportion of the effect of training on the dependent variables besides the independent effect. Indeed, regressions where resilience is removed from the models (not tabulated) show slightly larger effects of training on the dependent variables. However, this potential mediation effect was not significant (all *p* >.05) when tested using structural equation models and maximum likelihood estimation (results not tabulated). Overall, while there is a significant association between resilience and training levels in psychosomatic medicine, both variables have a much stronger independent contribution to explaining the four outcome measures as presented in Table 3.

### Qualitative results

Most respondents answered the open questions concerning challenges during the COVID-19 pandemic (*n* = 194, 84.7%) and their most important resources (*n* = 192, 83.8%). Results of QDA regarding physician-reported challenges and their most important resources during the COVID-19 crisis are presented in Tables 5 and 6.

**Table 5:** QDA of physician-reported challenges during the COVID-19 pandemic (*n* = 194)

Most difficult challenges during the COVID-19 pandemic			
**Category name**	**Description**	**Codings in QDA**	**n (%)**
Total		748	194 (100)
**Patient care**	Problems with patient care (divided in subcategories)	190	124 (63.9)
***Patient contact***	Restriction of contact in person, reduction in quality of patient contact, restricted communication	47	43 (22.2)
***Telemedicine***	Problems due to telemedical setting	44	43 (22.2)
***Other***	Other problems in patient care	35	30 (15.5)
***System***	Absent or restricted patient care within the health care system and related “collateral damages”	34	29 (14.9)
**Fear**	Patients‘ fears of the virus and associated problems in care	30	30 (15.5)
**Lack of information / uncertainty**	Lack of information, unsecurity, uncertainty, also information overload	71	64 (33.0)
**Coordination and administration**	Difficulties in coordination, administration and practice management	48	46 (23.7)
**Protective equipment**	Lack of protective equipment, problems acquiring prot. equipment	44	43 (22.2)
**Fear of infection**	Physician’s fear of being infected with the coronavirus, fear of being put into quarantine	34	33 (17.0)
**Leadership**	Difficulties in leading others or with leaders; team conflicts	27	24 (12.4)
**Financial burden**	Financial worries, loss of turnover	22	22 (11.3)
**Lack of work**	Restricted possiblities to work, closed practices, short-time work, too much time, restricted number of patients	18	17 (8.8)
**Lack of time**	Shortage of time, difficulties in management of time	18	16 (8.2)
**Job / Family**	Problems in reconciliation of work and family life	15	14 (7.2)
**Personal challenges**	Personal and emotional challenges of the pandemic situation	13	13 (6.7)
**Fear of infecting others**	Fear of infecting others with the coronavirus	11	11 (5.7)
**Hygiene measures**	Difficulties in implementing hygiene measures (e.g. keeping distances)	9	9 (4.6)
**Masks**	Compulsary masks, working with masks over a long period of time	8	8 (4.1)
**Management of tests**	Handling of tests and quarantine	8	6 (3.1)
**Skepticism regarding measures**	Being sceptical regarding government measures, being discontent with measures	7	7 (3.6)
**Other**	Other problems, not assignable to categories	6	6 (3.1)
**Lack of interaction**	Lack of interaction, social isolation	5	5 (2.6)

**Table 6:** QDA of physician-reported resources during the COVID-19 pandemic (*n* = 192)

Most helpful resources during the COVID-19 pandemic			
**Category name**	**Description**	**Codings in QDA**	**n (%)**
Total		736	192 (100)
**Team / collegues**	Professional relationships and exchange (colleagues, employees, team)	106	96 (50)
**Family**	Support from family, partnership, or close friends	57	55 (28.6)
**Resilience / Emotion regulation**	Keeping positive emotions, optimism, sedateness	50	44 (22.9)
**Experience / information**	High experience (with difficult situations), ability to gather specialist information	43	39 (20.3)
**Telemedicine**	Advantages of telemedicine, e.g. virtual platforms, prescriptions,…	39	33 (17.2)
**Personal coping stragies**	Personal coping strategies for recovery, e.g. sports, hobbies, living situation	34	28 (14.6)
**Job / financial situation**	Security regarding own job and/or financial situation	29	29 (15.1)
**Institution / superiors**	Support from superior organizations (professional organizations, media,…)	29	27 (14.1)
**Patients**	Gratefulness of patients, positive feedback, good doctor-patient relationships	25	25 (13.0)
**(Psy-)training**	Strategies from scecific PSY-training, that are experienced helpful	22	22 (11.5)
**Structure**	Clarity of structures and working processes	19	16 (8.3)
**Protective equipment**	Enough protective equipment, being supported by others (professional organizations, patients, companies) in this matter	18	18 (9.4)
**Time**	More free time, more time for patient contact, lower number of patients	18	16 (8.3)
**Meaning / appreciation**	Experiencing meaning due to the job, recognition by the public, feeling of being needed	13	13 (6.8)
**Autonomy**	Autonomy regarding treatment options or regarding own attitude	10	10 (5.2)
**Other**	Other resources, not assignable to categories	9	9 (4.7)
**Humor**	Humor	7	7 (3.6)
**Homeoffice / short-time work**	Possibility of short-time work and/or home office	6	6 (3.1)
**Religion**	Religion, belief	5	5 (2.6)
**Supervision**	Therapy, self-awareness, supervision, continuing education	5	5 (2.6)

#### Self-reported challenges and resources of physicians with high and low resilience

Comparisons of challenges and resources regarding resilience levels were based on a median split of the sample (Median = 41.0, Range = 29–50). Participants with a resilience sum score of 42 or more were considered to have “high resilience” (*n* = 45) whereas those with a value of 41 or less were considered to have “low resilience” (*n* = 55).

Physicians with low vs. high resilience were compared regarding their reported challenges and resources. Chi-square tests revealed that, within the mentioned challenges, there were no statistically significant differences between physicians with high or low resilience.

**Fig. 1:**
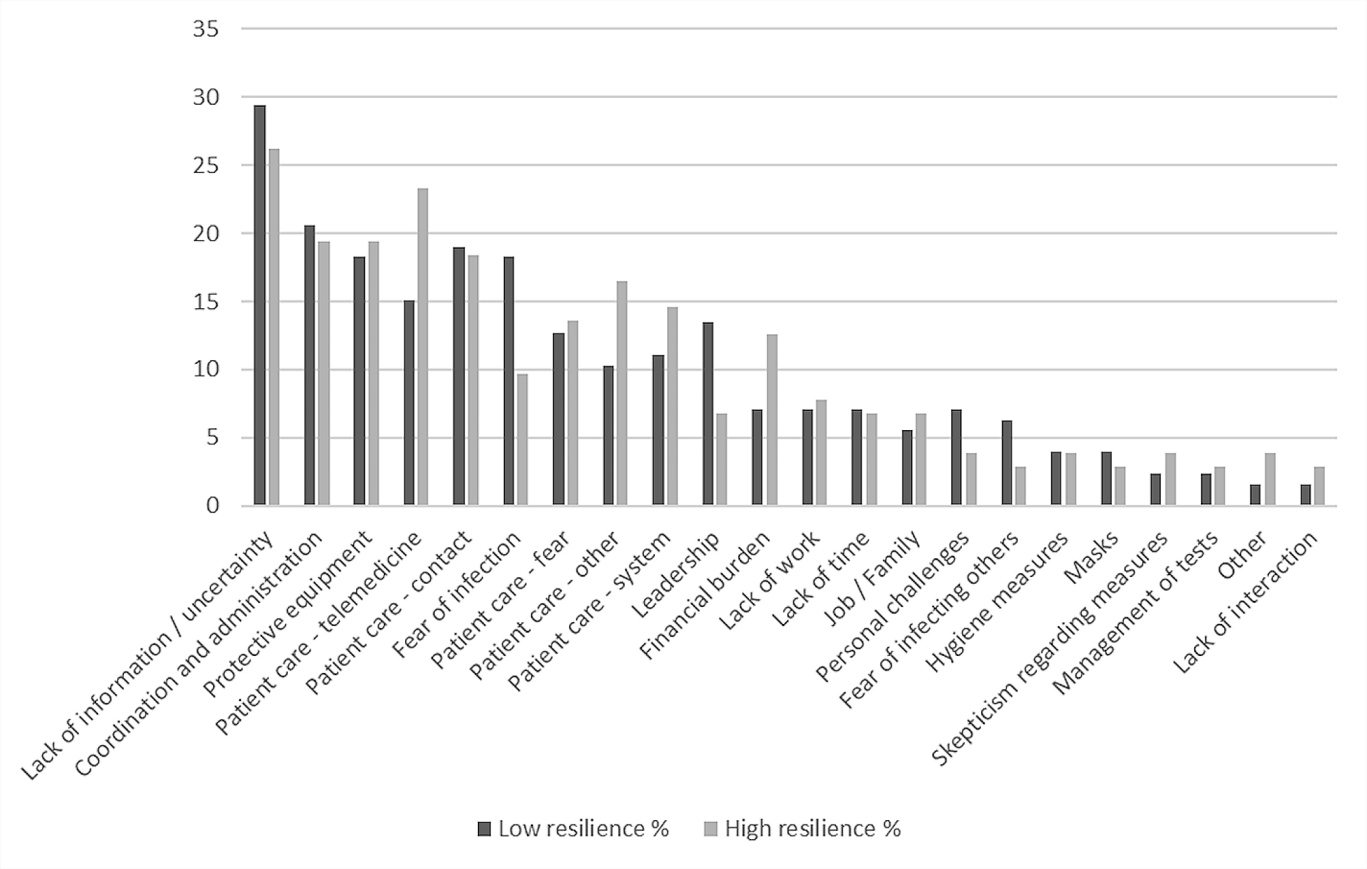
QDA frequency analysis of self-reported challenges according to resilience level

**Fig. 2:**
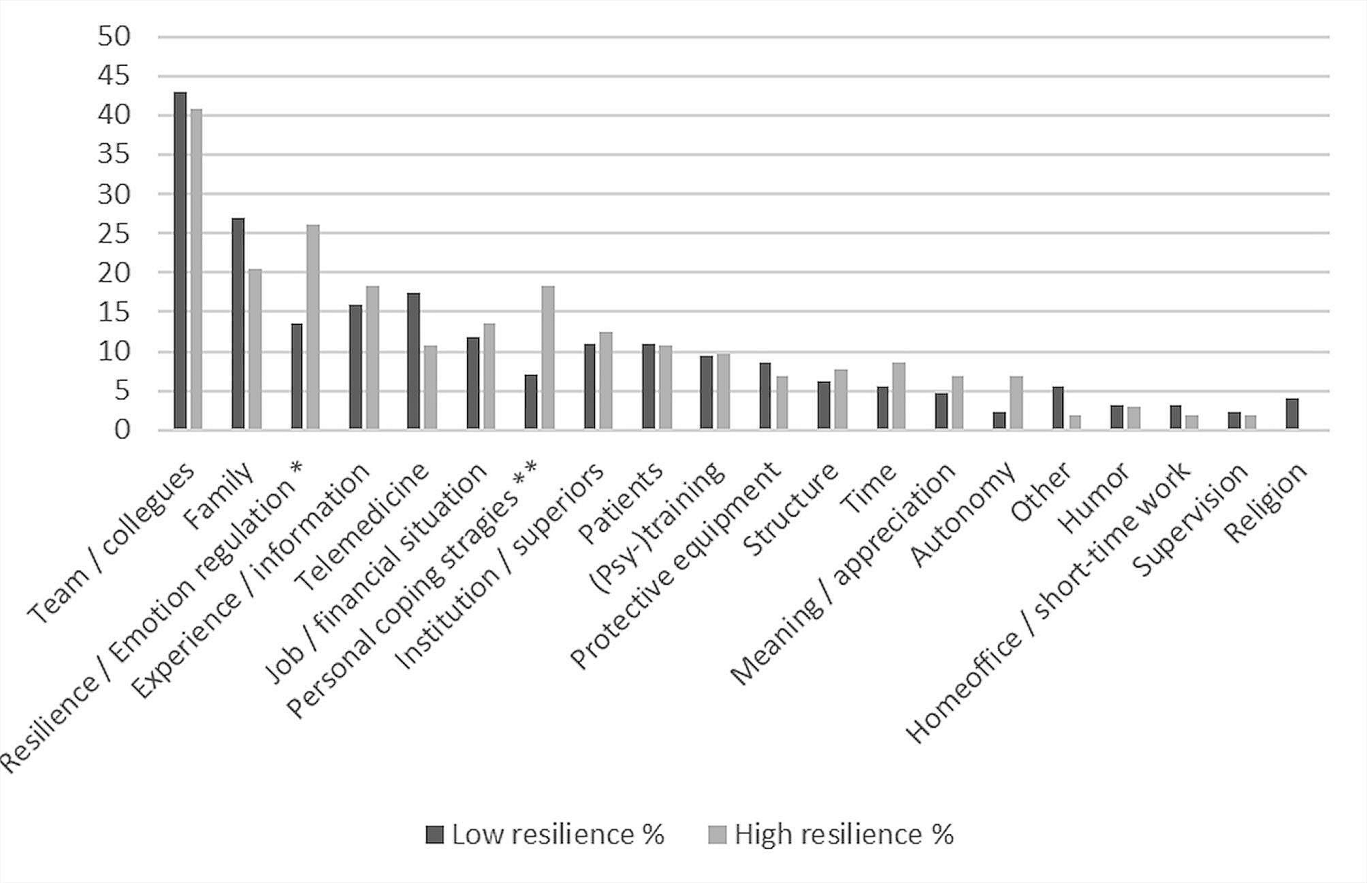
QDA frequency analysis of self-reported resources according to resilience level. *p <.05, **p <.01

As the naming of the most helpful resources is concerned, physicians with high vs. low resilience differed in the categories *resilience/emotion regulation* (Chi-square = 5.909, *p* =.015) and *personal coping strategies* (Chi-square = 6.747, *p* =.009): physicians with high resilience scores more often reported to employ strategies of emotion regulation and to have personal coping strategies (e.g. hobbies and sports). Figures 1 and 2 show the categories of QDA of self-reported difficulties and resources by comparing physicians with high vs. low resilience values.

#### Gender differences regarding self-reported challenges and resources

Gender differences regarding self-reported challenges and resources were calculated using Chi-square tests and revealed that males and females experienced similar challenges and resources during the COVID-19 pandemic. No significant differences were found when comparing the number of male and female physicians reporting on specific categories (*p* >.05).

## Discussion

Promoting resilience is recognized as a key strategy in medicine to counteract burnout and its known negative effects on health care providers, patients, and health care quality [3, 8, 39]. Recently, it has been shown that resilience has mitigated burnout symptoms triggered by the COVID-19 pandemic in *health care workers* [23]. To our knowledge, however, data about the association of resilience among *physicians* with perceived quality of delivered care during health care crisis have been scanty. In the present study, we investigated this assumed association in a specific cohort of medical doctors during the COVID-19 pandemic [6, 7]. More specifically, we looked at whether and, if so, to what extent resilience and postgraduate training in psychosomatic medicine in Austria were related to perceived quality of care and to further indicators of health care quality, i.e., professional autonomy, adequate time for patient care, and job satisfaction.

We found that both resilience and training level in psychosomatic medicine separately predicted perceived quality of delivered care when controlling for age, gender, number of patients, treatment of COVID-19 patients, own health concerns, financial strains, as well as training and resilience as control variables. Resilience also predicted professional autonomy and job satisfaction but not adequate time for patients during the COVID-19 pandemic. These results are in line with the sparse literature suggesting that physician resilience is associated with maintaining high quality of care during times of crisis [21, 22]. Similarly, a recent study among oncology professionals also reported a protective role of resilience on well-being and job performance during the COVID-19 pandemic [40].

In our study, resilience predicted three of four indicators of health care quality, with adequate time for patients being the exception– a result likely influenced by “systemic” conditions due to the COVID-19 pandemic. While the other indicators of health care quality - quality of care, professional autonomy, and job satisfaction– may be influenced by internal factors such as resilience, adequate time for patient care may depend more on external, organizational and institutional factors. These results imply that personal characteristics of individual physicians such as resilience promote more competence in dealing with challenging professional tasks in biopsychosocial medicine, particularly during times of pronounced health care crises.

The findings of the qualitative part of this study substantiate the main quantitative findings. Physicians with high resilience scores reported employing strategies of emotion regulation more often when asked to indicate three things that helped them most in their professional practice during the COVID-19 crisis. The following verbatim quotes answering this question give an insight into physician’s resources which were categorized as “resilience and emotion regulation”: “my adaptability to unfamiliar circumstances”; “I become creative and alert in critical situations and deal with patients in a calming way”; “my calm manner”; “my basically optimistic attitude”; “my ability of self-reflection”. Physicians with high resilience scores also reported more frequently relying on personal coping strategies such as cultivating hobbies and practicing sports. Examples for this category “personal coping strategies” are: “almost daily short hikes”; “regular movement with my children and dog”; “mindfulness meditation”; “good and healthy food”; “regeneration in the evening with good Netflix series”; “manual work at home”.

Moreover, the amount of training in psychosomatic medicine was also positively associated with the perceived quality of care and job satisfaction. Psychosomatic training may thus foster physicians` positive coping behavior with challenging professional situations. Similar positive effects by long-term trainings in psychosomatic medicine in Austria were reported before including effects on increased empathy for patients, the provision of adequate time for patient encounters, an increase in interdisciplinary cooperation with mental health professionals and improved job satisfaction after the training as compared to before the training [41–43].

Alongside the independent contribution of resilience and training background to quality of care, we found that more training was also associated with higher resilience levels. Although we cannot postulate causal relationships, we tested a potential mediation effect of the training background on quality of care through increased resilience, i.e., training in psychosomatic medicine might have increased physicians’ capacity to deal with and recover from stressful events, which in turn promoted quality of care. Although we did not find a significant effect on this matter, a potential mediation effect of resilience on the reported relationship between postgraduate training background and quality of care may deserve further attention, particularly in programs explicitly promoting resilience.

Successful interventions to promote individual physician resilience most likely require a medium- to long-term perspective. Although the Austrian PSY-curricula do not explicitly target an increase of resilience, they include several components that may be helpful in doing so, such as supervision, self-reflection and self-regulation skills [24, 43]. Taking into account the long duration of psychosomatic training and the slow rise in resilience mean scores over training levels (means from PSY-1 to PSY-4 are M = 4.02; M = 4.04; M = 4.13 and M = 4.23, respectively) we assume that it may indeed be difficult to foster resilience by short-term trainings as is often expected [44, 45]. Resilience, as a complex psychological construct with many influencing factors, is strongly connected to personal growth and traits, which are generally slow-changing factors [46], and a best practice guidance on how to improve resilience among physicians is still lacking [47]. The results of this study could contribute to inspire future designs of resilience trainings. Supervision, self-reflection, and self-regulation skills, including support for the successful management of difficult patient situations as a medical professional [1, 2, 39] may constitute important aspects of the psychosomatic training to foster resilience in medical doctors.

As demonstrated by the COVID-19 crisis, individual resilience of health care workers is linked to institutional resilience and work-related stressors [48, 49]. Therefore, long-term trainings for individual resilience in combination with interventions for institutional resilience and a reduction of stressors will be needed to ensure healthy work environments and sustainable quality of care.

This study combines quantitative and qualitative data collected during a particularly challenging period of time for physicians in Austria and can thus be regarded an important contribution to research on the highly relevant relationships between quality of care, resilience, and training background.

It seems plausible to assume that the reported results on physician resilience predicting perceived quality of care, professional autonomy and job satisfaction can also be generalized to other health care systems, yet, further research in other health care settings should substantiate this assumption. However, the generalizability of reported findings on training background in psychosomatic medicine as a predictor of quality of care appears to be limited to health care systems offering comparable training programs, which cover psychosomatic medicine and provide supervision, self-reflection and training in self-regulation skills to a similar or higher extent [50]. In addition, there are other limitations to be considered when interpreting the results of this study. First, this study presents a cross-sectional design and does not include real longitudinal measurements in assessing the three-time points. Therefore, estimates for outcome variables before and during the lockdown may be retrospectively biased. Nevertheless, the study was able to collect relevant data despite the rapid onset of the COVID-19 pandemic and the resulting changes in physicians’ work.

Secondly, the response rate of potential participants was lower than expected. From altogether 2792 Austrian doctors with psychosomatic training background, only 229 participated in the study. This may have been largely due to the heterogeneity of invitations for study participation, as some of the cooperating medical chambers sent out personal invitations while others informed on the study only by a newsletter. We also assume that many medical doctors were saturated in terms of survey participation, as many COVID-related studies were conducted at that time. As with all voluntary surveys, results may be prone to selection bias. For example, physicians more sensitive to biopsychosocial aspects of their profession may have been more likely to respond to the survey invitation. This may have contributed to the comparatively large proportion of study participants trained in psychotherapeutic medicine. Due to the low response rate and a potential selection bias, the reported results may not be transferable to the total group of medical doctors trained in psychosomatic medicine.

Third, findings in this study cohort may not apply to the same extent to medical specialists in other health care fields. Aiming to investigate the relevance of resilience and potential resilience-related training, we focused on the specific subgroup of medical doctors with a certified training in psychosomatic medicine, potentially limiting the scope of our findings. While psychosomatic training cannot be directly equated with resilience training, we are confident that at least parts of the training curricula directly relate to resilience-relevant skills and that the chosen population was thus suitable for our research questions.

As another limitation, this study reports on perceived quality of care and does not include objective quality indicators.

Finally, the study lacks a control group. Although the inclusion of a matched control group would have strengthened the study, time constraints prohibited recruiting a well-matched control group from all medical fields represented in the study population. Priority was thus given to launching the survey while the immediate effects of the COVID-19 pandemic and the first lockdown in Austria were still salient for practitioners.

## Conclusion

Physician resilience and training level in psychosomatic medicine are both independently and significantly associated with perceived quality of patient care and job satisfaction in times of a health care crisis. Promotion of resilience among health-care workers may require long-term trainings and interventions in which institutional and individual resilience interventions should complement each other.

### Electronic supplementary material

Below is the link to the electronic supplementary material.

Supplementary Material 1

Supplementary Material 2

## Data Availability

Data of the quantitative and qualitative parts of the study will be provided upon request by the corresponding author.
