# Supplementary material for: Physician resilience and perceived quality of care among medical doctors with training in psychosomatic medicine during the COVID-19 pandemic: a quantitative and qualitative analysis
Source: BMC Health Serv Res. 2024 Feb 27;24:249. doi: 10.1186/s12913-024-10681-1 (PMC10900785; doi:10.1186/s12913-024-10681-1)
Supplement: Supplementary file 1 — Supplementary Material 1 [file 12913_2024_10681_MOESM1_ESM.docx]

**Additional File 1: Survey**

1. **General information and professional background**

Please indicate how old you are:

☐ under 30 years

☐ 30-39 years

☐ 40-49 years

☐ 50-59 years

☐ 60-69 years

☐ over 69 years old

Please enter your gender:

☐ female

☐ male

☐ various

Are you currently professionally active as a doctor?

☐ yes

☐ no

What medical subject do you work in?

☐ General medicine

☐ Gynecology and obstetrics

☐ Internal medicine; internal special subjects

☐ Pediatric and adolescent medicine

☐ Child and adolescent psychiatry and psychotherapeutic medicine; Child and adolescent psychiatry

☐ Neurology; Neurology and Psychiatry

☐ Psychiatry and psychotherapeutic medicine; Psychiatry; Psychiatry and Neurology

☐ Other subject: ____________________ (optional)

☐ Other: ______________________ (optional)

Which of the additional training courses mentioned have you acquired? (multiple selection possible)

☐ ÖÄK-PSY-1 diploma

☐ ÖÄK-PSY-2 diploma/specialization in subject-specific psychosomatic medicine

☐ ÖÄK-PSY-3 diploma

☐ Further additional training/s that deal with psychological aspects of health:

__________________________________

Do your colleagues/employees at your workplace know that you hold an ÖÄK-PSY diploma or a specialization in specialist psychosomatic medicine?

☐ I work alone, so the question cannot be answered for me.

☐ yes

☐ no

☐ don't know

Do you work (primarily) as an employee or as a self-employed person?

☐ independent

☐ employed

[If independent = YES]:

How do you work in self-employment?

☐ as a panel doctor

☐ as an optional doctor

☐ in another form: ___________________________

[If independent = YES]:

How many employees do you have?

______________

[If employed = YES]:

Where do you primarily carry out your current work as an employee?

☐ Hospital/clinic

☐ Outpatient care

☐ Practice

☐ Public service

☐ Rehabilitation center

☐ In another area: ______________________

Approximately what percentage of your total working time usually goes to direct patient care?

____%

Please indicate approximately how many hours per week you have worked or are working for the periods listed.

Before the Covid-19 crisis: _____ hours per week

During the lockdown (mid-March to the end of April): from___to __ hours per week

Last week: _____ hours per week

Please indicate approximately how many patients per week you have looked after or are looking after for the periods listed.

Before the Covid-19 crisis: _____ patients per week

During the lockdown (mid-March to the end of April): from___to __ patients per week

Currently: _____ patients per week

1. **Handling of difficult situations**

German 10-item-version of the Connor-Davidson Resilience Scale (CD-RISC)

1. **Health status/stresses of the patients**

Please assess the **physical** and **mental health status** as well as the **social situation** of your patients
during the lockdown and currently **compared to “before the COVID-19 crisis”**.

If this does not seem possible to you, please mark: “No statement possible.”

|  | worse | | same | | | better | | no statement possible |
| --- | --- | --- | --- | --- | --- | --- | --- | --- |
|  | -3 | -2 | -1 | 0 | +1 | +2 | +3 |  |
| How do you assess the **physical** health of your patients during the lockdown compared to before the COVID-19 crisis? | ☐ | ☐ | ☐ | ☐ | ☐ | ☐ | ☐ | ☐ |
| How do you assess the current **physical** health of your patients in comparison to before the COVID-19 crisis? | ☐ | ☐ | ☐ | ☐ | ☐ | ☐ | ☐ | ☐ |
| How do you assess the **mental** health of your patients during the lockdown compared to before the COVID-19 crisis? | ☐ | ☐ | ☐ | ☐ | ☐ | ☐ | ☐ | ☐ |
| How do you assess the current **mental** health status of your patients in comparison to before the COVID-19 crisis? | ☐ | ☐ | ☐ | ☐ | ☐ | ☐ | ☐ | ☐ |
| How do you assess the **social situation** of your patients during the lockdown compared to before the COVID-19 crisis? | ☐ | ☐ | ☐ | ☐ | ☐ | ☐ | ☐ | ☐ |
| How do you assess the current **social situation** of your patients compared to before the COVID-19 crisis? | ☐ | ☐ | ☐ | ☐ | ☐ | ☐ | ☐ | ☐ |

1. **Medical practice**

Please assess to what extent the following statements apply to you for the specified periods (before the COVID-19 crisis, during the lockdown, at the present time). Please provide a response for each statement (from 0 “strongly disagree” to 4 “strongly agree”).

Response scale: 0 strongly disagree

1 disagree

2 neutral

3 agree

4 strongly agree

I had/have adequate time to spend with my patients during a typical patient visit.

|  | Strongly disagree | | | Strongly agree | | |
| --- | --- | --- | --- | --- | --- | --- |
|  | 0 | 1 | 2 | | 3 | 4 |
| Before the COVID-19 crisis | ☐ | ☐ | ☐ | | ☐ | ☐ |
| During the lockdown (mid March until the end of April) | ☐ | ☐ | ☐ | | ☐ | ☐ |
| At the present time | ☐ | ☐ | ☐ | | ☐ | ☐ |

I had/have the freedom to make clinical decisions that meet my patients’ needs.

|  | Strongly disagree | | | Strongly agree | | |
| --- | --- | --- | --- | --- | --- | --- |
|  | 0 | 1 | 2 | | 3 | 4 |
| Before the COVID-19 crisis | ☐ | ☐ | ☐ | | ☐ | ☐ |
| During the lockdown (mid March until the end of April) | ☐ | ☐ | ☐ | | ☐ | ☐ |
| At the present time | ☐ | ☐ | ☐ | | ☐ | ☐ |

It was/is possible for me to provide high quality care to all of my patients.

|  | Strongly disagree | | | Strongly agree | | |
| --- | --- | --- | --- | --- | --- | --- |
|  | 0 | 1 | 2 | | 3 | 4 |
| Before the COVID-19 crisis | ☐ | ☐ | ☐ | | ☐ | ☐ |
| During the lockdown (mid March until the end of April) | ☐ | ☐ | ☐ | | ☐ | ☐ |
| At the present time | ☐ | ☐ | ☐ | | ☐ | ☐ |

I could/can apply the skills acquired in the PSY training in my daily work.

|  | Strongly disagree | | | Strongly agree | | |
| --- | --- | --- | --- | --- | --- | --- |
|  | 0 | 1 | 2 | | 3 | 4 |
| Before the COVID-19 crisis | ☐ | ☐ | ☐ | | ☐ | ☐ |
| During the lockdown (mid March until the end of April) | ☐ | ☐ | ☐ | | ☐ | ☐ |
| At the present time | ☐ | ☐ | ☐ | | ☐ | ☐ |

**Job satisfaction**

On the whole, how satisfied were you/are you with your job in the specified periods?

Antwortskala 0 very dissatisfied

1 dissatisfied

2 neither satisfied nor dissatisfied

3 satisfied

4 very satisfied

|  | Very dissatisfied | | | Very satisfied | | |
| --- | --- | --- | --- | --- | --- | --- |
|  | 0 | 1 | 2 | | 3 | 4 |
| Before the COVID-19 crisis | ☐ | ☐ | ☐ | | ☐ | ☐ |
| During the lockdown (mid March until the end of April) | ☐ | ☐ | ☐ | | ☐ | ☐ |
| At the present time | ☐ | ☐ | ☐ | | ☐ | ☐ |

**Protective equipment**

Did you have enough protective equipment available **during** the lockdwon?

☐ yes

☐ rather yes

☐ rather no

☐ no

Do you **currently** have enough protective equipment available?

☐ yes

☐ rather yes

☐ rather no

☐ no

**Telemedicine**

Have you used telemedicine methods (e.g. telephone, video telephony, specialized software application) **during the lockdown**?

☐ Never

☐ Rarely

☐ Sometimes

☐ Often

☐ Always

Are you **currently** using telemedicine methods (e.g. telephone, video telephony, specialized software applications)?

☐ Never

☐ Rarely

☐ Sometimes

☐ Often

☐ Always

**COVID-19**

Did you treat patients who were SARS-CoV-2 positive during the lockdown (mid-March to the end of April)?

☐ yes

☐ no

☐ unsecure

How many people you know personally have tested positive for SARS-CoV-2?

____ persons

Have you been worried about your own health during the COVID-19 lockdown?

☐ Yes

☐ Rather yes

☐ Rather no

☐ No

Are you currently concerned about your health due to COVID-19?

☐ Yes

☐ Rather yes

☐ Rather no

☐ No

**General conditions of medical activity**

Please rate to what extent you agree or disagree with the following statements. Please provide a response for each statement (from 0 “Strongly disagree” to 6 “Strongly agree”).

Response scale: 0 strongly disagree

1 disagree

2 rather disagree

3 neither nor

4 rather agree

5 agree

6 strongly agree

|  | Strongly disagree | | |  | Strongly agree | | |
| --- | --- | --- | --- | --- | --- | --- | --- |
|  | 0 | 1 | 2 | 3 | 4 | 5 | 6 |
| I am receiving sufficient support from those responsible in the healthcare system in connection with the COVID-19 crisis. | ☐ | ☐ | ☐ | ☐ | ☐ | ☐ | ☐ |
| Personally, I am currently in a financially stressful situation. | ☐ | ☐ | ☐ | ☐ | ☐ | ☐ | ☐ |
| I believe that the economy in Austria will have recovered in a year. | ☐ | ☐ | ☐ | ☐ | ☐ | ☐ | ☐ |

Please indicate the three most difficult job-related challenges during the COVID-19 crisis.

______________________________________________________________________________________

Please indicate three things that helped you most in your professional practice during the COVID-19 crisis.

______________________________________________________________________________________

Final remarks/notes (optional)

______________________________________________________________________________________

**Thank you very much for your participation!**
