# Supplementary material for: Physician resilience and perceived quality of care among medical doctors with training in psychosomatic medicine during the COVID-19 pandemic: a quantitative and qualitative analysis
Source: BMC Health Serv Res. 2024 Feb 27;24:249. doi: 10.1186/s12913-024-10681-1 (PMC10900785; doi:10.1186/s12913-024-10681-1)
Supplement: Supplementary file 2 — Supplementary Material 2 [file 12913_2024_10681_MOESM2_ESM.docx]

**Additional File 2: Training levels in psychosomatic medicine and differences in time periods**

Additional Table 1 shows means and standard deviations of all variables by PSY-Curricula training levels and by time period where applicable. Besides our main outcome variables of interest (quality of care, professional autonomy, time for patients, and job satisfaction), we report means of concerns about one’s health due to COVID-19 during the lockdown (health concerns), whether the participants had treated COVID-19 patients, financial strain as perceived at the time of completing the survey, the percentage of working time spent on patient care (patients), age (measured in 6 categories from under 30 to over 69, see sociodemographic characteristics in Table 1) and gender.

Means indicate a positive linear trend in the outcome variables and in resilience with higher psychosomatic training score, i.e., higher training levels are generally associated with higher levels in resilience and with quality of care, professional autonomy, time for patients and job satisfaction regardless of the time period. However, as indicated by the mean values in all four outcome variables, participants generally indicated a decline during the lockdown (see Additional Table 1, Column “Total”).

Simple repeated measures ANOVAs (not tabulated) with quality of care, professional autonomy, time for patients, and job satisfaction as dependent variables and the three time periods as independent factor reveal that for all four variables, the time periods differ significantly from each other (all p < .05). Using t-tests with “present” as the reference category, we find that the time before the lockdown was perceived as significantly better, and during the lockdown as significantly worse for variables quality of care, professional autonomy, and job satisfaction (all p < .05). Regarding time for patients, we only find a significant difference between the period during the lockdown, which was perceived as significantly worse (p < .05). Analyses of potential interaction effects between time and training as well as resilience are explored below.

**Additional Table 1**

|  | **Postgraduate training levels in psychosomatic medicine** | | | | | | | | | | |  | | |
| --- | --- | --- | --- | --- | --- | --- | --- | --- | --- | --- | --- | --- | --- | --- |
|  | PSY-1  (*n*=21) | |  | PSY-2  (*n*=74) | |  | PSY-3  (*n*=106) | |  | PSY-4  (*n*=28) | |  | Total  (*N*=229) | |
| **Variables** | *M* | *SD* |  | *M* | *SD* |  | *M* | *SD* |  | *M* | *SD* |  | *M* | *SD* |
| Resilience | 4.02 | 0.41 |  | 4.04 | 0.46 |  | 4.13 | 0.45 |  | 4.23 | 0.43 |  | 4.10 | 0.45 |
| Quality  of care |  |  |  |  |  |  |  |  |  |  |  |  |  |  |
| Before | 3.90 | 1.30 |  | 4.35 | 0.77 |  | 4.56 | 0.76 |  | 4.71 | 0.46 |  | 4.45 | 0.82 |
| During | 3.14 | 1.31 |  | 3.22 | 1.33 |  | 3.29 | 1.40 |  | 4.00 | 1.05 |  | 3.34 | 1.35 |
| Present | 3.90 | 1.04 |  | 4.16 | 0.79 |  | 4.26 | 0.99 |  | 4.57 | 0.57 |  | 4.24 | 0.90 |
| Profesional Autonomy |  |  |  |  |  |  |  |  |  |  |  |  |  |  |
| Before | 4.29 | 0.90 |  | 4.30 | 0.82 |  | 4.43 | 0.76 |  | 4.57 | 0.63 |  | 4.39 | 0.78 |
| During | 3.00 | 1.22 |  | 3.27 | 1.26 |  | 3.33 | 1.34 |  | 4.21 | 1.10 |  | 3.39 | 1.31 |
| Present | 4.10 | 0.94 |  | 4.03 | 0.83 |  | 4.16 | 0.93 |  | 4.43 | 0.88 |  | 4.14 | 0.89 |
| Time for patients |  |  |  |  |  |  |  |  |  |  |  |  |  |  |
| Before | 3.62 | 1.28 |  | 3.80 | 1.13 |  | 4.17 | 0.99 |  | 4.43 | 0.79 |  | 4.03 | 1.07 |
| During | 4.24 | 1.14 |  | 3.80 | 1.23 |  | 3.65 | 1.44 |  | 4.25 | 1.08 |  | 3.83 | 1.32 |
| Present | 3.86 | 1.06 |  | 3.78 | 1.14 |  | 4.08 | 1.04 |  | 4.39 | 0.79 |  | 4.00 | 1.06 |
| Job satisfaction |  |  |  |  |  |  |  |  |  |  |  |  |  |  |
| Before | 4.29 | 0.78 |  | 4.36 | 0.82 |  | 4.41 | 0.93 |  | 4.75 | 0.52 |  | 4.42 | 0.85 |
| During | 3.10 | 1.41 |  | 3.42 | 1.37 |  | 3.57 | 1.29 |  | 4.29 | 1.08 |  | 3.56 | 1.33 |
| Present | 3.67 | 1.11 |  | 4.05 | 1.02 |  | 4.24 | 0.97 |  | 4.57 | 0.84 |  | 4.17 | 1.00 |
| Health concerns ^b^ | 2.67 | 1.02 |  | 2.24 | 1.07 |  | 2.25 | 0.98 |  | 2.46 | 0.96 |  | 2.31 | 1.01 |
| Treated Cov.  patients (proportion) ^b^ | 0.29 | 0.46 |  | 0.36 | 0.48 |  | 0.23 | 0.42 |  | 0.18 | 0.39 |  | 0.27 | 0.45 |
| Financial strain ^b^ | 1.60 | 0.99 |  | 2.04 | 1.12 |  | 2.16 | 1.24 |  | 2.18 | 1.39 |  | 2.07 | 1.21 |
| Patients (% of work) | 72.86 | 23.64 |  | 74.96 | 19.60 |  | 69.79 | 23.50 |  | 72.68 | 22.01 |  | 72.10 | 22.11 |
| Age group | 3.43 | 0.68 |  | 3.57 | 0.83 |  | 4.15 | 0.98 |  | 4.32 | 0.90 |  | 3.92 | 0.95 |
| Female (proportion) | 0.76 | 0.44 |  | 0.66 | 0.48 |  | 0.70 | 0.46 |  | 0.71 | 0.46 |  | 0.69 | 0.46 |

^b^… lower sample size (N=214).

To explore how the three time periods influenced our results from the regressions on the participant means, we conducted repeated measures ANOVAs (see Additional Table 2). We entered the same variables as in our main regressions (see Table 3), with the addition of time period as a three-level factor using the original values for each dependent variable, resulting in 642 observations from 214 participants. Due to the rANOVAs’ specifications, the main results (i.e., the between-subject effects) remained unchanged. However, we found significant interactions between time period and psychosomatic training for professional autonomy and for time for patients.

Additional Figures 1 and 2 illustrate these interactions, presenting the mean values of professional autonomy and time for patients by time period and psychosomatic training (dichotomized into low and high training groups). Participants with low psychosomatic training indicated a steeper decline in autonomy during the lockdown. Conversely, starting from a lower baseline, they indicated a slightly positive development regarding time for patients during the lockdown as opposed to the negative perception of high-training participants (see Additional Table 1 for the original mean values).

**Additional Table 2**

|  | Quality  of care *F* | Professional  autonomy *F* | Time for  patients *F* | Job  satisfaction  *F* | *df*  (num., denom.) |
| --- | --- | --- | --- | --- | --- |
| Period | 0.61 | 0.10 | 1.01 | 0.60 | 2, 422 |
| Training | 5.92* | 3.42 | 2.54 | 6.65* | 1, 205 |
| Resilience | 7.48** | 4.84* | 0.87 | 11.88*** | 1, 205 |
| Control variables |  |  |  |  |  |
| Health concerns | 0.07 | 0.44 | 0.75 | 3.40 | 1, 205 |
| Treated Cov.  Patients | 4.61* | 3.71 | 10.72** | 7.40** | 1, 205 |
| Financial strain | 8.60** | 5.92* | 1.83 | 10.78** | 1, 205 |
| Patients | 0.48 | 0.23 | 0.02 | 0.55 | 1, 205 |
| Age | 2.41 | 0.63 | 0.02 | 2.79 | 1, 205 |
| Gender | 1.85 | 0.06 | 0.21 | 1.12 | 1, 205 |
| Interactions |  |  |  |  |  |
| Period*Training | 0.19 | 4.03* | 3.43* | 2.67 | 2, 422 |
| Period*Resilience | 0.52 | 2.14 | 0.71 | 0.50 | 2, 422 |

This table presents results from repeated measures ANOVAs with Type III sum of squares and Satterthwaite’s method. Each column shows *F* values and significance levels for one of the four dependent variables. The DF column presents the degrees of freedom, which are identical for each of the four models. For each dependent variable, three values per participant entered the model, resulting in 642 observations from 214 participants. The sample size is reduced because health concerns and financial strain are included as independent variables and were completed by fewer participants. *… p < .05, **… p < .01, ***… p < .001.

**Additional Figure 1**


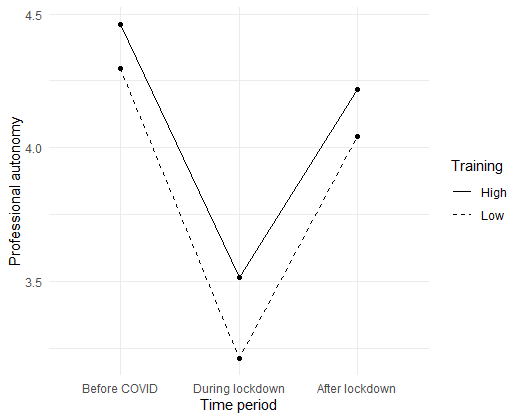


Mean values of professional autonomy by time period and psychosomatic training. Low training subsumes psychosomatic training levels 1 and 2, high 3 and 4 (see Additional Table 1 for the original means).

**Additional Figure 2**


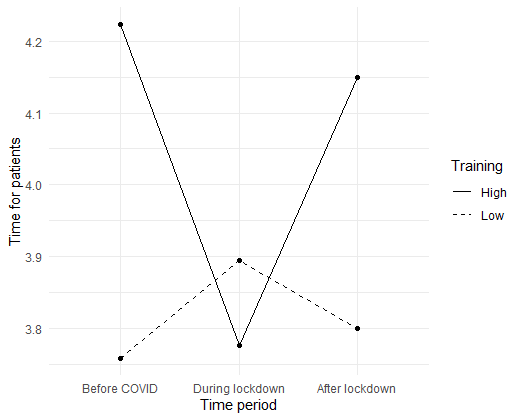


Mean values of time for patients by time period and psychosomatic training. Low training subsumes psychosomatic training levels 1 and 2, high 3 and 4 (see Additional Table 1 for the original means).
